# Supplementary material for: Synthesis of Estrone Heterodimers and Evaluation of Their In Vitro Antiproliferative Activity
Source: Int J Mol Sci. 2024 Apr 12;25(8):4274. doi: 10.3390/ijms25084274 (PMC11050183; doi:10.3390/ijms25084274)

Synthesis of Estrone Heterodimers and Evaluation of Their In Vitro Antiproliferative Activity

Characterization data of the reported heterodimers (**11–14**)

3-[{1-(3-benzyloxy-17β-hydroxy-13α-estra-1,3,5(10)-trien-16α-yl)-1H-1,2,3-triazol-4-yl}methoxy]-13α-hydroxymethyl-14β-propyl-des-D-estra-1,3,5(10)-triene (**11**)

11 was obtained as a white solid (159 mg, 87%). Mp 104–108 °C, R_f = 0.80^A. Anal. Calcd. For $C_{47}H_{59}N_3O_4$: C, 77.33; H, 8.15. Found: C, 77.41; H, 8.20. ^{1}H NMR δ ppm ($CDCl_3$): 0.77 and 1.28 (2xs, 2x3H, 18-H_3 and 18′-H_3); 0.92 (t, 1H, *J* = 6.8 Hz, 16a′-H_3); 2.80–2.85 (overlapping multiplets, 2x2H, 6-H_2 and 6′-H_2); 3.33 and 3.51 (2xm, 2x1H, 17′-H_2); 4.14 (d, 1H, *J* = 8.1 Hz, 17-H); 4.74 (m, 1H, 16-H); 5.03 (s, 2H, benzyl-OCH_2); 5.17 (s, 2H, OCH_2); 6.69 and 6.71 (2xd, 2x1H, *J* = 2.2 Hz, 4-H and 4′-H); 6.78 and 6.81 (2xdd, 2x1H, *J* = 8.6 Hz, *J* = 2.2 Hz, 2-H and 2′-H); 7.16 and 7.21 (2xd, 2x1H, *J* = 8.6 Hz, 1-H and 1′-H); 7.32 (t, 1H, *J* = 7.3 Hz, 4″-H); 7.38 (t, 2H, 3″- and 5″-H), 7.43 (d, 2H, *J* = 7.3 Hz, 2″- and 6″-H); 7.70 (s, 1H, C=CH), ^{13}C NMR δ ppm 14.7 and 16.0 and 28.7 (C-18 and C-18′ and C-16a′); 25.0; 26.4; 26.9; 27.4; 27.5; 28.9; 30.2; 30.7; 31.2; 31.9; 35.6; 37.4; 38.7; 41.0; 41.7; 42.1; 43.5; 45.2; 48.6; 62.0 (OCH_2); 66.1 (C-16); 69.9 (benzyl-OCH_2); 71.3 (C-17′); 85.2 (C-17); 112.4 and 113.0 (C-2 and C-2′); 114.2 and 114.3 (C-4 and C-4′); 122.4 (C=$\underline{\text{C}}$H); 126.6 and 127.7 (C-1 and C-1′); 127.4 (2C: C-2″ and C-6″); 127.8 (C-4″); 128.5 (2C: C-3″ and C-5″); 133.5 and 133.8 (C-10 and C-10′); 137.2 (C-1″); 137.5 and 138.1 (C-5 and C-5′); 144.1 ($\underline{\text{C}}$=CH); 156.1 and 156.6 (C-3 and C-3′).
ESI-HRMS: m/z: 730.45636 $[M+H]^+$ ($C_{47}H_{60}N_3O_4$ requires 730.45783 $[M+H]^+$).

3-[{1-(3-benzyloxy-17α-hydroxy-13α-estra-1,3,5(10)-trien-16β-yl)-1H-1,2,3-triazol-4-yl}methoxy]-13α-hydroxymethyl-14β-propyl-des-D-estra-1,3,5(10)-triene (**12**)

12 was obtained as a white solid (171 mg, 94%). Mp 98–102 °C, R_f = 0.76^A. Anal. Calcd. For $C_{47}H_{59}N_3O_4$: C, 77.33; H, 8.15. Found: C, 77.43; H, 8.21. ^{1}H NMR δ ppm ($CDCl_3$): 0.77 and 1.26 (2xs, 2x3H, 18-H_3 and 18′-H_3); 0.91 (t, 1H, *J* = 6.8 Hz, 16a′-H_3); 3.33 and 3.51 (2xd, 2x1H, *J* = 10.9 Hz, 17′-H_2); 4.52 (d, 1H, *J* = 8.2 Hz, 17-H); 4.77 (m, 1H, 16-H); 5.03 (s, 2H, benzyl-OCH_2); 5.16 (s, 2H, OCH_2); 6.71(2xd, 2x1H, *J* = 2.2 Hz, 4-H and 4′-H); 6.78 (2xdd, 2x1H, *J* = 8.6 Hz, *J* = 2.2 Hz, 2-H and 2′-H); 7.20 (2xd, 2x1H, *J* = 8.6 Hz, 1-H and 1′-H); 7.31 (t, 1H, *J* = 7.3 Hz, 4″-H); 7.38 (t, 2H, 3″- and 5″-H), 7.42 (d, 2H, *J* = 7.3 Hz, 2″- and 6″-H); 7.66 (s, 1H, C=CH), ^{13}C NMR δ ppm 14.7 and 16.0 and 23.0 (C-18 and C-18′ and C-16a′); 25.0; 26.4; 26.5; 27.4; 28.3; 30.3; 30.7; 31.2; 31.7; 33.1; 35.6; 38.7; 41.7; 42.0; 42.9; 43.2; 43.5; 45.2; 48.3; 62.0 (OCH_2); 66.4 (C-16); 69.9 (benzyl-OCH_2); 71.3 (C-17′); 78.7 (C-17); 112.3 and 112.7 (C-2 and C-2′); 114.3 and 114.6 (C-4 and C-4′); 122.6 (C=$\underline{\text{C}}$H); 126.6 and 126.9 (C-1 and C-1′); 127.4 (2C: C-2″ and C-6″); 127.8 (C-4″); 128.5 (2C: C-3″ and C-5″); 132.0 and 133.4 (C-10 and C-10′); 137.2 and 138.1 and 138.2 (C-1″ and C-5 and C-5′); 144.3 ($\underline{\text{C}}$=CH); 156.1 and 156.8 (C-3 and C-3′). ESI-HRMS: m/z: 730.45668 $[M+H]^+$ ($C_{47}H_{60}N_3O_4$ requires 730.45783 $[M+H]^+$).

3-[{1-(3-benzyloxy-17β-hydroxy-13α-estra-1,3,5(10)-trien-16α-yl)-1H-1,2,3-triazol-4-yl}methoxy]-14β-propyl-des-D-estra-1,3,5(10)-trien-13α-carbaldehyde oxime (**13**)

13 was obtained as a white solid (167 mg, 90%). Mp 108–110 °C, R_f = 0.73^B. Anal. Calcd. For $C_{47}H_{58}N_4O_4$: C, 75.98; H, 7.87. Found: C, 76.04; H, 7.96. ^{1}H NMR δ ppm (DMSO-d_6): 0.83 (t, 3H, *J* = 6.8 Hz, 16a′-H_3); 0.98 and 1.17 (2xs, 2x3H, 18-H_3 and 18′-H_3); 2.72–2.80 (overlapping multiplets, 4H, 6-H_2 and 6′-H_2); 3.92 (t, 1H, *J* = 6.3 Hz, 17-H); 4.84 (m, 1H, 16-H); 5.04 (s, 2H, benzyl-OCH_2); 5.07 (m, 2H, OCH_2); 5.26 (d, 1H, *J* = 5.6 Hz, OH); 6.68 and 6.73 (2xd, 2x1H, *J* = 2.2 Hz, 4-H and 4′-H); 6.77–6.79 (overlapping multiplets, 2H, 2-H and 2′-H); 7.14 and 7.19 (2xd, 2x1H, *J* = 8.6 Hz, 1-H and 1′-H); 7.18 (s, 1H, 17′-H), 7.31 (t, 1H, *J* = 7.3 Hz, 4″-H); 7.38 (t, 2H, *J* = 7.3 Hz, 3″- and 5″-H), 7.42 (d, 2H, *J* = 7.3 Hz, 2″- and 6″-H); 8.24 (s, 1H, C=CH); 10.38 (s, 1H, OH), ^{13}C NMR δ ppm (DMSO-d_6): 14.3 and 15.4 and 28.7 (C-16a′ and C-18 and C-18′); 23.8; 25.6; 26.7; 27.1; 28.1, 28.2; 29.6; 29.8; 31.5; 32.0; 37.1; 37.5; 40.1; 40.5; 40.6; 41.7; 42.7; 46.9; 48.4; 60.9 (OCH_2); 66.2 (C-16); 68.9 (benzyl-OCH_2); 84.3 (C-17); 112.3 and 112.7 (C-2 and C-2′); 113.7 and 113.8 (C-4 and C-4′); 123.6 (C=$\underline{\text{C}}$H); 126.3 and 127.3 (C-1 and C-1′); 127.4 (2C, C-2″ and C-6″); 127.6 (C-4″); 128.3 (2C, C-3″ and C-5″); 132.1 and 133.5 (C-10 and C-10′); 137.3 (2C) and 137.5 (C-1″ and C-5 and C-

5′); 142.5 (C=CH); 155.8 and 155.9 (C-3 and C-3′); 157.8 (C-17′). ESI-HRMS: m/z: 743.45197 [M+H]+ ($C_{47}H_{59}N_4O_4$ requires 743.45308 [M+H]+).

3-[{1-(3-benzyloxy-17α-hydroxy-13α-estra-1,3,5(10)-trien-16β-yl)-1H-1,2,3-triazol-4-yl}methoxy]-14β-propyl-des-D-estra-1,3,5(10)-trien-13α-carbaldehyde oxime (**14**)

14 was obtained as a white solid (165 mg, 89%). Mp 112–115 °C, R_f = 0.67[B]. Anal. Calcd. For $C_{47}H_{58}N_4O_4$: C, 75.98; H, 7.87. Found: C, 76.08; H, 7.95. ^{1}H NMR δ ppm (DMSO-d_6): 0.83 (t, 3H, *J* = 6.8 Hz, 16a′-H_3); 0.98 (overlapping singlets, 2x3H, 18-H_3 and 18′-H_3); 2.77 (overlapping multiplets, 4H, 6-H_2 and 6′-H_2); 4.35 (m, 1H, 17-H); 4.85 (m, 1H, 16-H); 5.05 (overlapping singlets, 4H, 2xOCH_2); 5.17 (m, 1H, OH); 6.71 (overlapping multiplets, 2H, 4-H and 4′-H); 6.77–6.80 (overlapping multiplets, 2H, 2-H and 2′-H); 7.17–7.24 (overlapping multiplets, 3H, 1-H, 17-H and 1′-H); 7.31 (t, 1H, *J* = 7.3 Hz, 4"-H); 7.38 (t, 2H, *J* = 7.3 Hz, 3"- and 5"-H), 7.42 (d, 2H, *J* = 7.3 Hz, 2"- and 6"-H); 8.21 (s, 1H, C=CH), 10.37 (s, 1H, OH). ^{13}C NMR δ ppm (DMSO-d_6): 14.9, 16.0 and 23.4 (C-16a′, C-18 and C-18′); 24.4; 26.2; 26.6; 27.3; 28.4; 30.4 (2C); 32.1; 32.6; 33.3; 37.7; 41.1; 41.2; 42.1; 42.8; 43.2; 43.3; 47.5; 48.0; 61.4 (OCH_2); 66.0 (C-16); 69.5 (benzyl-OCH_2); 77.6 (C-17); 112.9 and 113.0 (C-2 and C-2′); 114.5 and 114.8 (C-4 and C-4′); 124.8 (C=CH); 126.9 and 127.3 (C-1 and C-1′); 127.9 (2C, C-2″ and C-6″); 128.1 (C-4″); 128.9 (2C, C-3″ and C-5″); 132.6 and 132.7 (C-10 and C-10′); 137.9 (2C) and 138.3 (C-5, C-5′ and C-1″); 143.1 (C=CH); 156.4 and 156.7 (C-3 and C-3′); 158.4 (C-17′).

ESI-HRMS: m/z: 743.45188 [M+H]+ ($C_{47}H_{59}N_4O_4$ requires 743.45308 [M+H]+).

^{1}H and ^{13}C NMR spectra of the heterodimers **11–14**

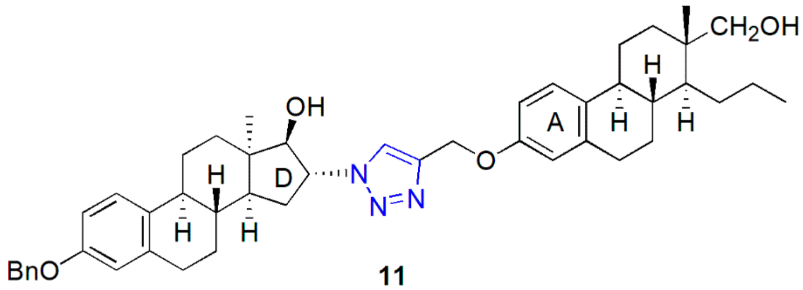

11

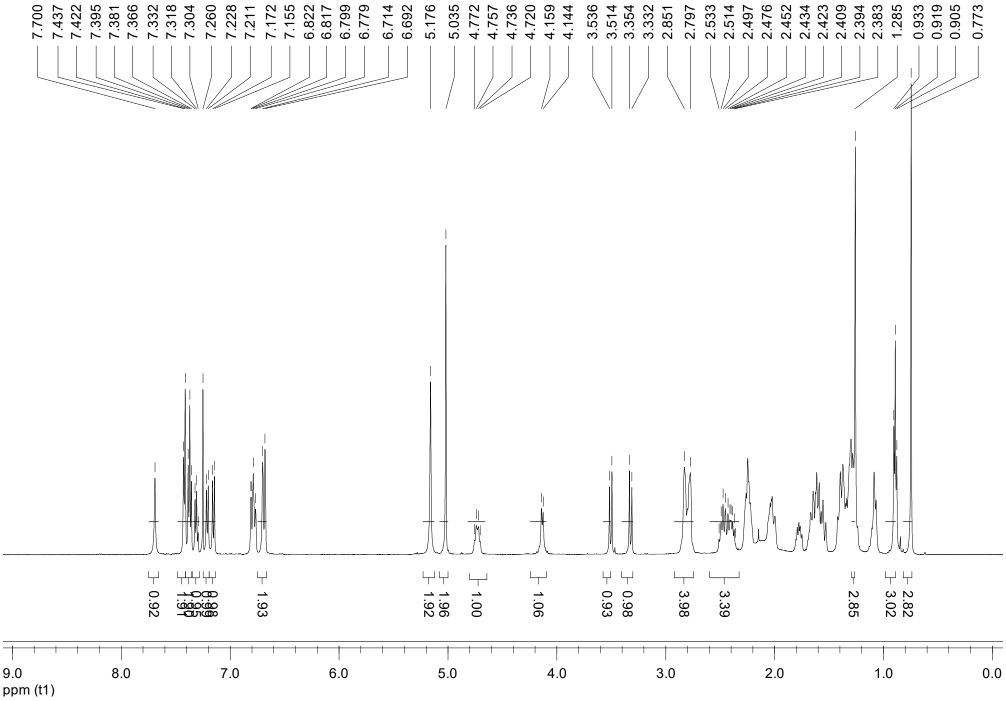

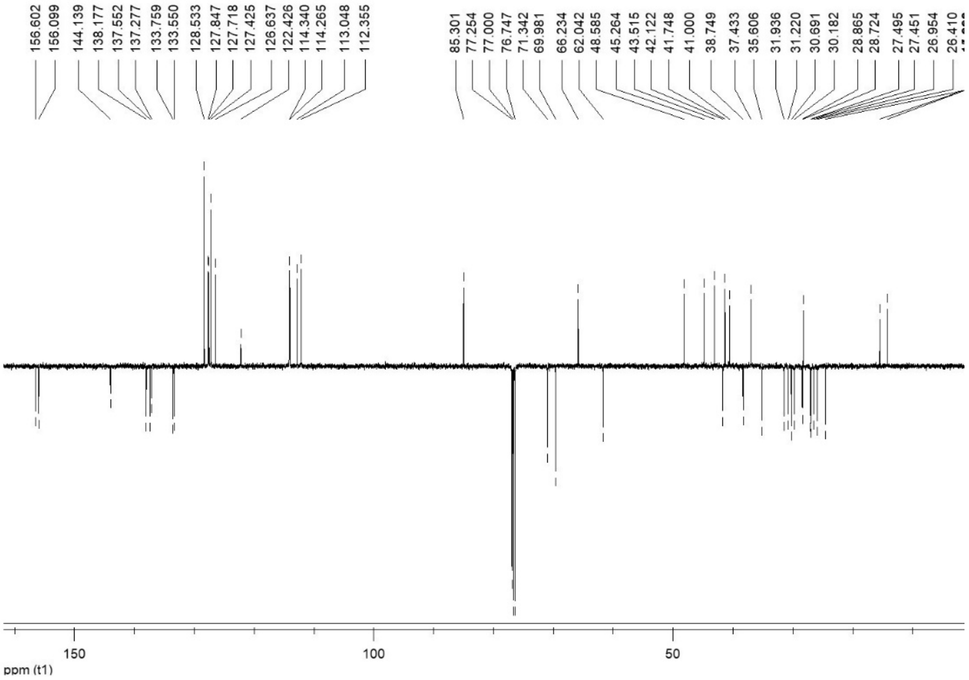

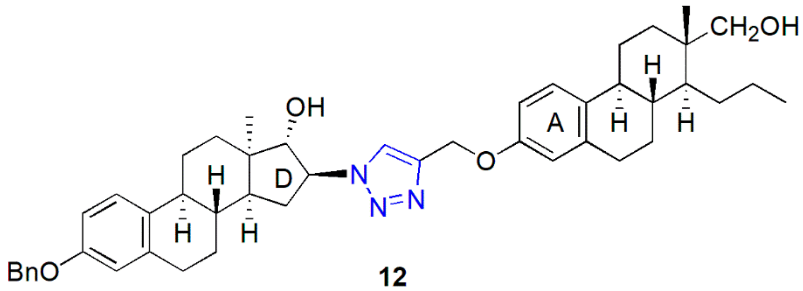

OH
D
H
H
H
BnO
N
N
N
O
A
H
H
H
CH2OH

12

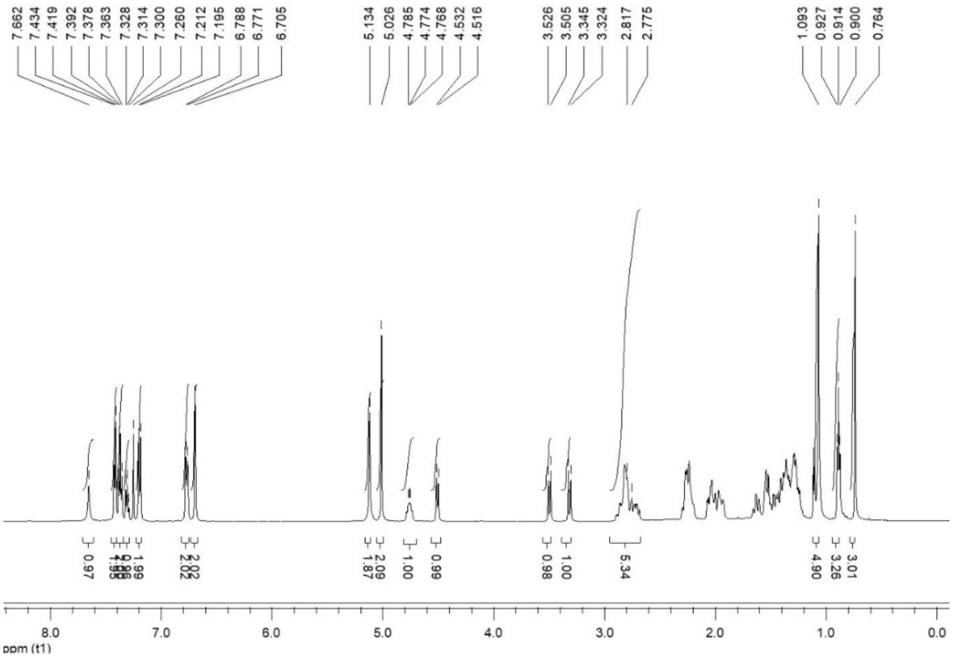

7.662
7.434
7.419
7.392
7.378
7.363
7.328
7.314
7.300
7.260
7.212
7.195
6.788
6.771
6.705
5.134
5.026
4.785
4.774
4.768
4.532
4.516
3.526
3.505
3.345
3.324
2.817
2.775
1.093
0.927
0.914
0.900
0.764
0.97
1.99
2.02
1.87
2.09
1.00
0.99
0.98
1.00
5.34
4.90
3.26
3.01
8.0
7.0
6.0
5.0
4.0
3.0
2.0
1.0
0.0
ppm (t1)

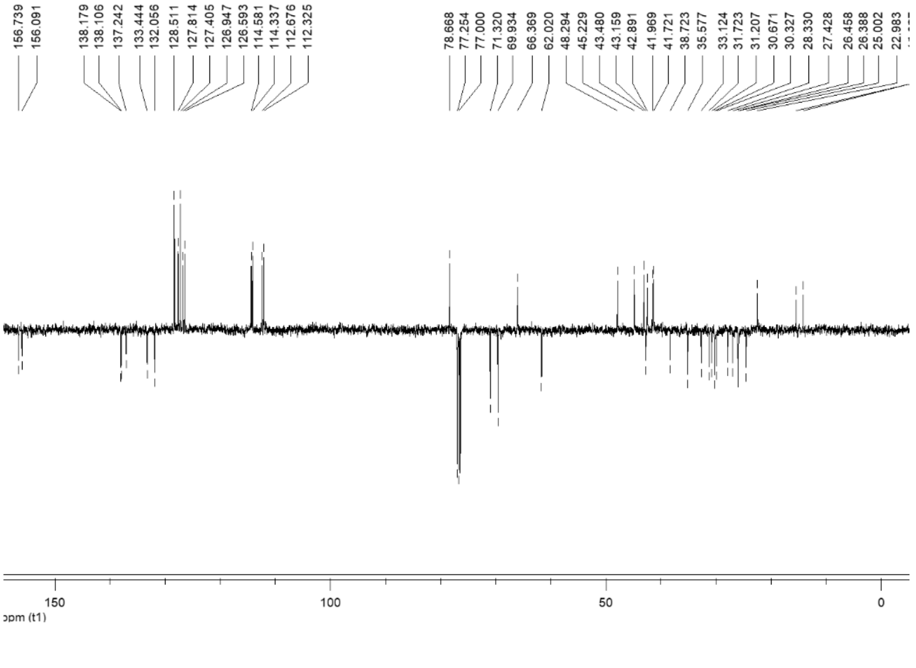

156.739
156.091
138.179
138.106
137.242
133.444
132.056
128.511
127.814
127.405
126.947
126.593
114.581
114.337
112.676
112.325
78.668
77.254
77.000
71.320
69.934
66.369
62.020
48.294
45.229
43.480
43.159
42.891
41.969
41.721
38.723
35.577
33.124
31.723
31.207
30.671
30.327
28.330
27.428
26.458
26.388
25.002
22.983
150
100
50
0
ppm (t1)

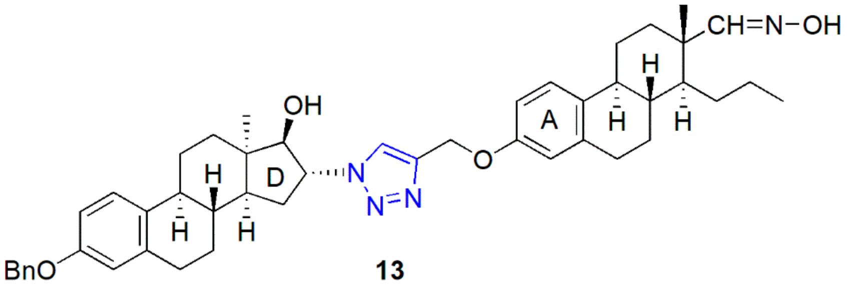
CH=N−OH
OH
H
H
H
D
A
H
H
H
BnO
O
N
N=N
13

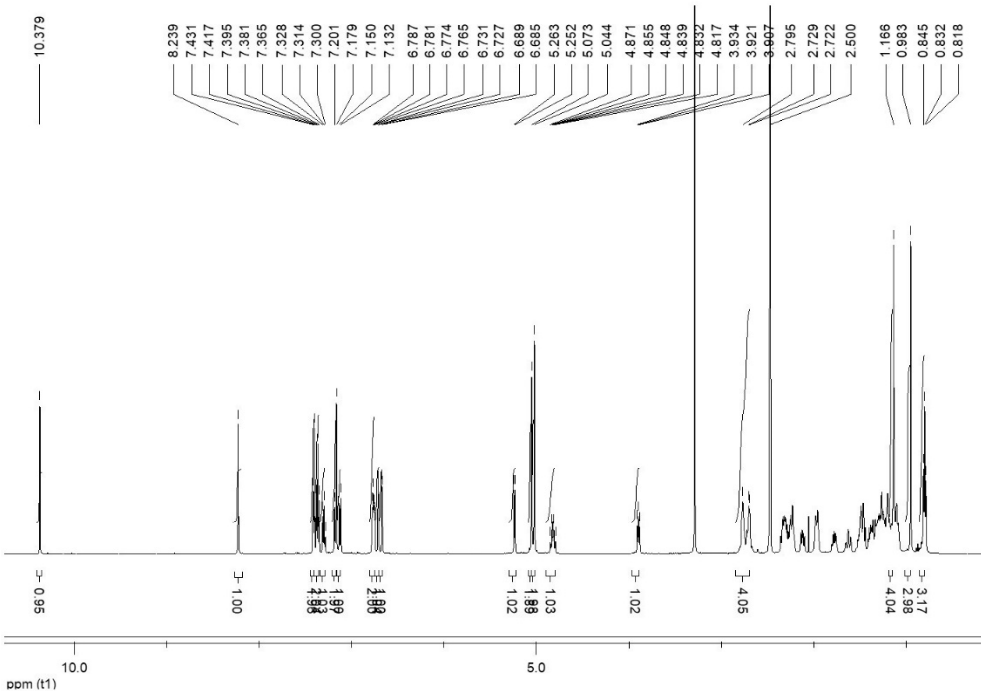
10.379
8.239
7.431
7.417
7.395
7.381
7.365
7.328
7.314
7.300
7.201
7.179
7.150
7.132
6.787
6.781
6.774
6.765
6.731
6.727
6.689
6.685
5.263
5.252
5.073
5.044
4.871
4.855
4.848
4.839
4.832
4.817
3.934
3.921
3.907
2.795
2.729
2.722
2.500
1.166
0.983
0.845
0.832
0.818
0.95
1.00
1.02
1.03
1.02
4.05
4.04
2.98
3.17
10.0
5.0
ppm (t1)

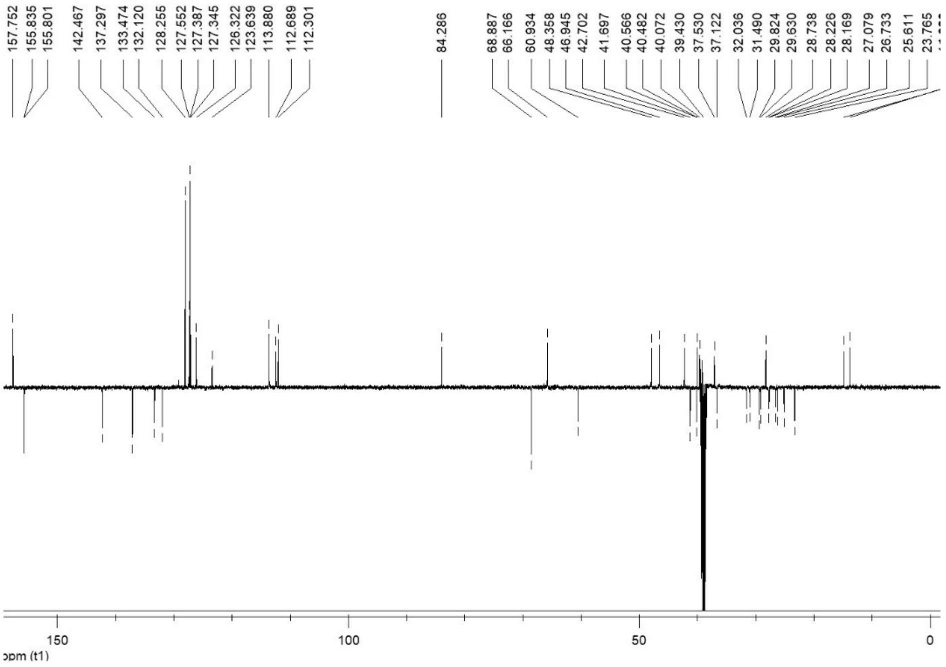
157.752
155.835
155.801
142.467
137.297
133.474
132.120
128.255
127.552
127.387
127.345
126.322
123.639
113.880
112.689
112.301
84.286
68.887
66.166
60.934
48.358
46.945
42.702
41.697
40.566
40.482
40.072
39.430
37.530
37.122
32.036
31.490
29.824
29.630
28.738
28.226
28.169
27.079
26.733
25.611
23.765
150
100
50
0
ppm (t1)

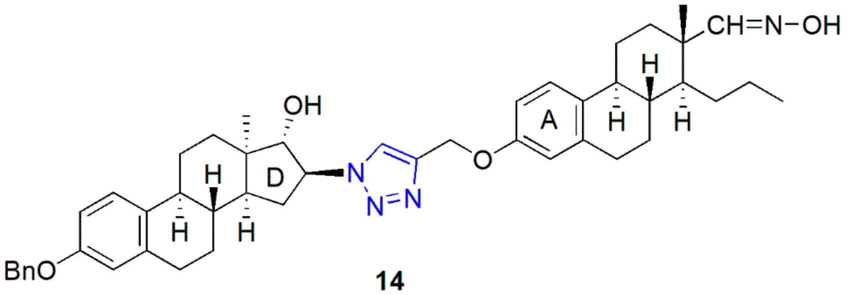
CH=N−OH
OH
BnO
N
N=N
O
A
D
H

14

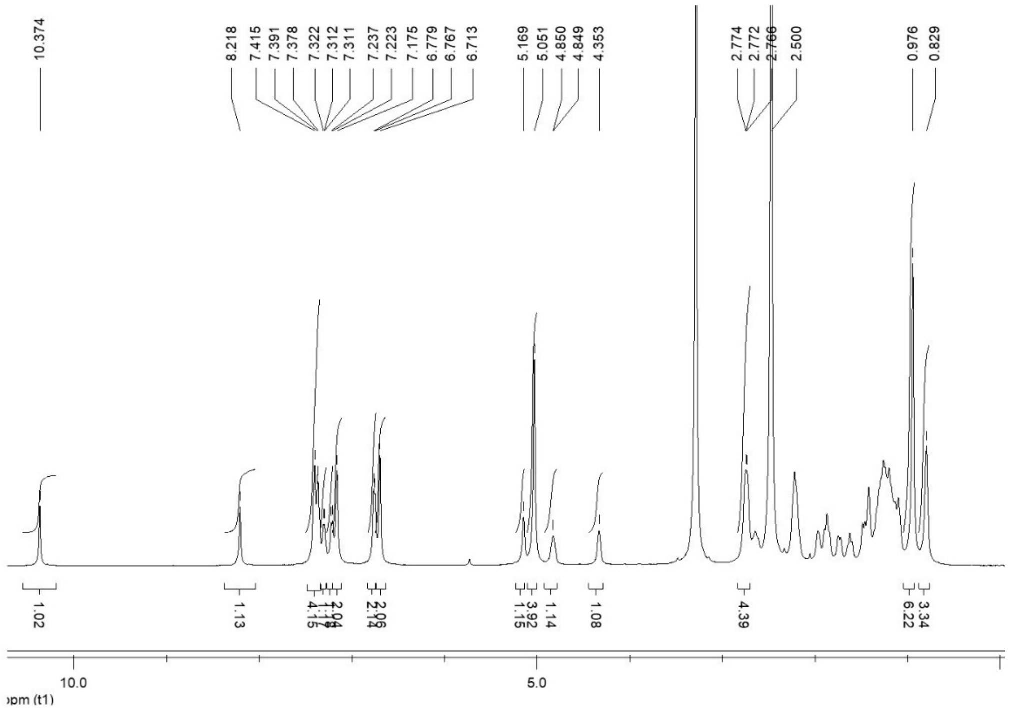
10.374
8.218
7.415
7.391
7.378
7.322
7.312
7.311
7.237
7.223
7.175
6.779
6.767
6.713
5.169
5.051
4.850
4.849
4.353
2.774
2.772
2.766
2.500
0.976
0.829
1.02
1.13
4.15
2.04
2.06
1.15
3.92
1.14
1.08
4.39
6.22
3.34
10.0
5.0
ppm (t1)

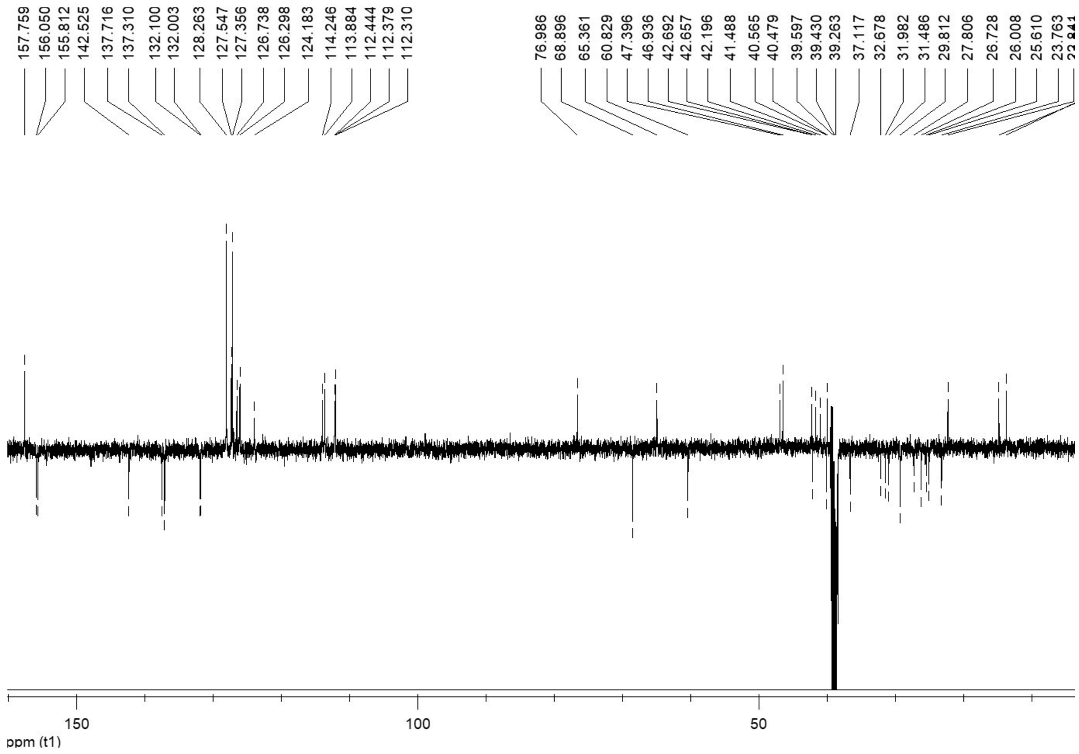
157.759
156.050
155.812
142.525
137.716
137.310
132.100
132.003
128.263
127.547
127.356
126.738
126.298
124.183
114.246
113.884
112.444
112.379
112.310
76.986
68.896
65.361
60.829
47.396
46.936
42.692
42.657
42.196
41.488
40.565
40.479
39.597
39.430
39.263
37.117
32.678
31.982
31.486
29.812
27.806
26.728
26.008
25.610
23.763
150
100
50
ppm (t1)

Figure S1. Ligand **12** in DMSO solution (a,) and water solution (b,)

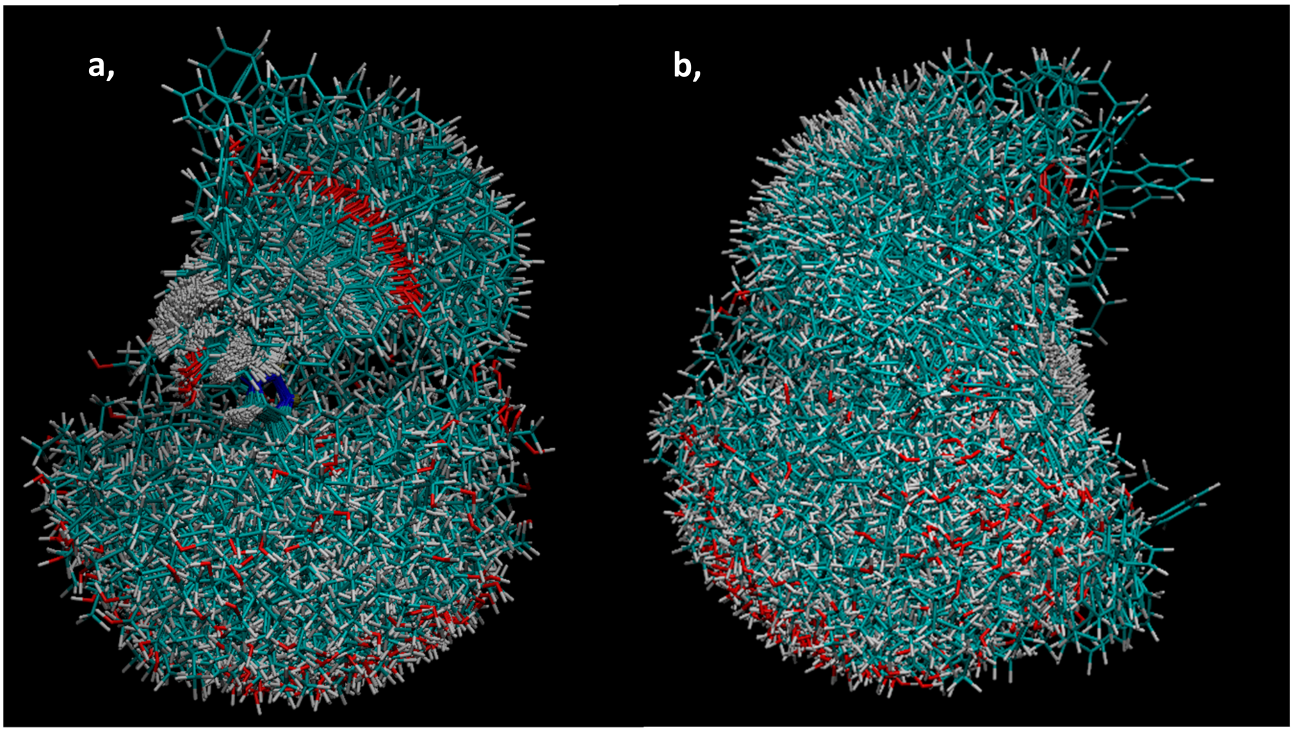

Figure S2. Ligand-Protein (LP) interactions along the 200 ns REST trajectory of compound **12** (closed sterane skeleton part in the binding pocket).

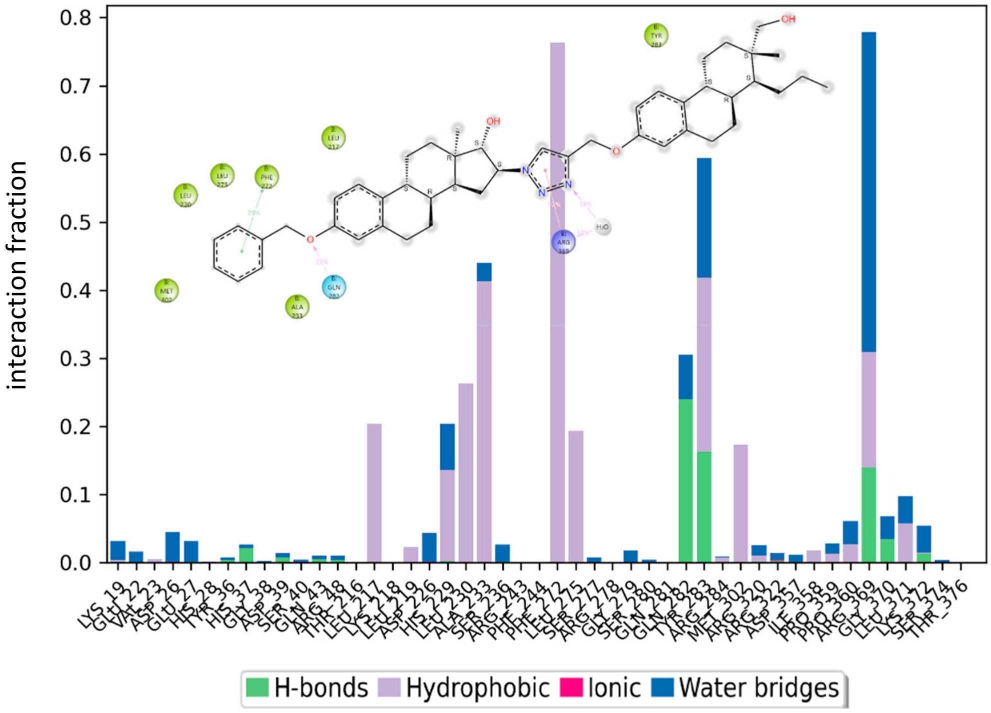

Figure S3. Ligand-Protein (LP) interactions along the 200 ns REST trajectory of compound **12** (open-steroid skeleton part in the binding pocket).

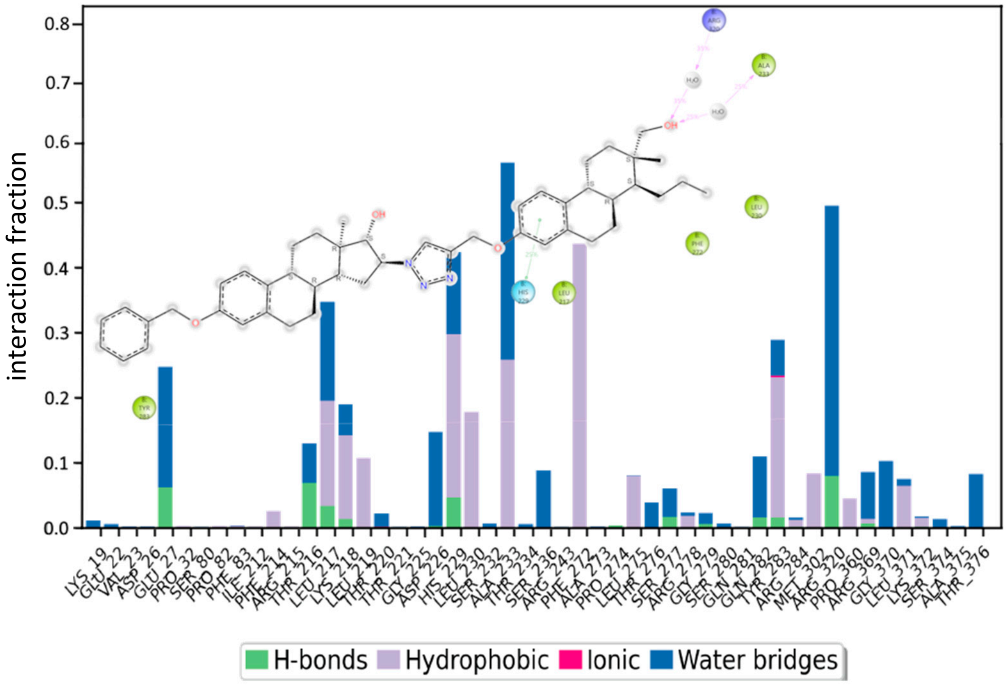

Supplement: Supplementary file 1 [file ijms-25-04274-s001.zip › ijms-2921545-supplementary.pdf]
